# Closed Genome Sequence of Phytopathogen Biocontrol Agent *Bacillus velezensis* Strain AGVL-005, Isolated from Soybean

**DOI:** 10.1128/genomeA.00057-18

**Published:** 2018-02-15

**Authors:** Victor Satler Pylro, Armando Cavalcante Franco Dias, Fernando Dini Andreote, Daniel Kumazawa Morais, Alessandro de Mello Varani, Cristiane Cipolla Fasanella Andreote, Eduardo Roberto de Almeida Bernardo, Tiago Zucchi

**Affiliations:** a Soil Microbiology Laboratory, Luiz de Queiroz College of Agriculture, University of São Paulo (ESALQ/USP), Piracicaba, Sao Paulo, Brazil; b Andrios Assessoria, Piracicaba, Sao Paulo, Brazil; c Institute of Microbiology, Czech Academy of Sciences (CAS), Prague, Czech Republic; d Departamento de Tecnologia, Faculdade de Ciências Agrárias e Veterinárias, Universidade Estadual Paulista (Unesp), Jaboticabal, Sao Paulo, Brazil; e Agrivalle Brasil Indústria e Comércio de Produtos Agrícolas Ltda., Salto, Sao Paulo, Brazil

## Abstract

We report here the closed and near-complete genome sequence and annotation of *Bacillus velezensis* strain AGVL-005, a bacterium isolated from soybean seeds in Brazil and used for phytopathogen biocontrol.

## GENOME ANNOUNCEMENT

Biological control using bacteria shows great potential as an eco-friendly and feasible alternative to replace, or at least diminish, the application of chemicals for pathogen control (1). Understanding the mechanisms involved in the interaction between any biological control agents and target phytopathogen is crucial to enhance and extend the use of these organisms in agriculture. To gain insight into the use of *Bacillus velezensis* strain AGVL-005 (a Gram-positive and rod-shaped soil bacterium) for the biological control of phytopathogens, we sequenced the genome using the MinION Mk1B device (MIN-101B; Oxford Nanopore Technologies, UK).

Briefly, ∼1 µg of unsheared genomic DNA was submitted to end repair and dA-tailing steps using the NEBNext Ultra end repair/dA-tailing module (New England BioLabs, USA) and then treated with the 1D Genomic DNA sequencing kit for the MinION device (catalog number SQK-LSK-108; Oxford Nanopore Technologies, UK). The resulting library was sequenced using a flow cell Spot-ON Mk1 (FLO-MIN 106 R9 version; Oxford Nanopore Technologies), with the R9 version library loading bead kit (EXP-LLB001; Oxford Nanopore Technologies). The raw reads were acquired using the MinKNOW software version 1.7.14 in a 48-h run experiment and base called using the Albacore software version 2.0.2.

A total of 810,820 reads were obtained, with sizes ranging from 31 to 69,980 bp in length. All reads were *de novo* assembled using Canu version 1.5 (2), using default parameters for Nanopore data. The genome was assembled into 14 contigs. Analysis based on the SIS software (3) and BLAST indicated that most of the contig ends were related to rRNA operon genes. A proposed genome consensus was manually closed by comparisons against the closest reference genome (*Bacillus amyloliquefaciens* subsp. *plantarum* strain FZB42 [GenBank accession number NC_009725]) and BLAST analysis. An improved consensus sequence for the draft assembly was obtained with the Nanopolish software version 0.7.2 (https://github.com/jts/nanopolish), using default parameters. Genome completeness and contamination were estimated using CheckM (4) in the lineage-specific mode. Genome statistics were estimated with QUAST (5) using *B. amyloliquefaciens* subsp. *plantarum* strain FZB42 as a reference genome. The average nucleotide identity based on BLAST (ANIb) between our genome and its reference was determined by JSpecies (6); ANI scores of >95% indicate that they belong to the same species (7, 8).

The final genome consists of a single contiguous circular chromosome. The estimated genome size is 4,146,154 bp, with a G+C content of 45.98%. The genome completeness estimated by CheckM was 90.66%, and the contamination was 0.64%, being classified as a near-complete and low-contaminated genome. Genome annotation was performed with PATRIC version 3.2.45beta (9). We identified 6,261 coding sequences (CDSs) and 92 predicted noncoding RNAs (71 tRNA and 21 rRNA). The calculated ANIb was 98.3% between *B. velezensis* AGVL-005 and *B. amyloliquefaciens* subsp. *plantarum* (reference strain), which classified them as belonging to the same species. It is important to highlight that *B. amyloliquefaciens* subsp. *plantarum* was recently classified as a later heterotypic synonym of *Bacillus velezensis* (10); therefore, our isolate was identified according to this reclassification.

### Accession number(s).

This whole-genome shotgun project has been deposited at DDBJ/EMBL/GenBank under the accession number CP024922. The version described in this paper is the first version, CP024922.
